# Breaking silence: a survey of barriers to goals of care discussions from the perspective of oncology practitioners

**DOI:** 10.1186/s12885-019-5333-x

**Published:** 2019-02-08

**Authors:** Katrina Lynn Piggott, Ameen Patel, Arthur Wong, Leslie Martin, Alexandra Patel, Matthew Patel, Yudong Liu, Sukhbinder Dhesy-Thind, John J. You

**Affiliations:** 1 Department of Geriatric Medicine, 30 Bond Street, Room 4-002, Shuter Wing, Toronto, ON M5B 1W8 Canada; 2 Department of Medicine, McMaster University, 1200 Main Street West, Hamilton, ON L8N 3Z5 Canada; 3 Department of Occupational Science and Occupational Therapy, The University of Toronto, 500 University Avenue, Toronto, ON M5G 1V7 Canada; 4 Royal College of Surgeons in Ireland, 123 St Stephen’s Green, Dublin 2, Ireland; 5 Schulich School of Dentistry, University of Western Ontario, 1151 Richmond St, London, ON N6A 5C1 Canada; 6 Department of Oncology, McMaster University, 699 Concession St, Hamilton, ON L8V 5C2 Canada; 7 Department of Health Research Methods, Evidence, and Impact, McMaster University, 1280 Main St West, Hamilton, ON L8S 4K1 Canada

**Keywords:** Goals of care discussions, End of life, Barriers, Cancer care

## Abstract

**Background:**

Cancer is the leading cause of death in the developed world, and yet healthcare practitioners infrequently discuss goals of care (GoC) with hospitalized cancer patients. We sought to identify barriers to GoC discussions from the perspectives of staff oncologists, oncology residents, and oncology nurses.

**Methods:**

This was a single center survey of staff oncologists, oncology residents, and inpatient oncology nurses. Barriers to GoC discussions were assessed on a 7-point Likert scale (1 = extremely unimportant; 7 = extremely important).

**Results:**

Between July 2013 and May 2014, of 185 eligible oncology clinicians, 30 staff oncologists, 10 oncology residents, and 28 oncology nurses returned surveys (response rate of 37%). The most important barriers to GoC discussions were patient and family factors. They included family members’ difficulty accepting poor prognoses (mean score 5.9, 95% CI [5.7, 6.2]), lack of family agreement in the goals of care (mean score 5.8, 95% CI [5.5, 6.1]), difficulty understanding the limitations of life-sustaining treatments (mean score 5.8, 95% CI [5.6, 6.1]), lack of patients’ capacity to make goals of care decisions (mean score 5.7, 95% CI [5.5, 6.0]), and language barriers (mean score 5.7, 95% CI [5.4, 5.9]). Participants viewed system factors and healthcare provider factors as less important barriers.

**Conclusions:**

Oncology practitioners perceive patient and family factors as the most limiting barriers to GoC discussions. Our findings underscore the need for oncology clinicians to be equipped with strong communication skills to help patients and families navigate GoC discussions.

**Electronic supplementary material:**

The online version of this article (10.1186/s12885-019-5333-x) contains supplementary material, which is available to authorized users.

## Background

Cancer is a leading cause of mortality in the developed world [1]. Most terminally-ill cancer patients prefer to die at home [2]. Despite this, approximately one third still die in hospital, and nearly 10 % die in the intensive care unit (ICU) [3]. An estimated 30 % of patients offered chemotherapy are in the last months of life, and this percentage is increasing each year, as are emergency room (ER) visits, hospitalizations, and admissions to the ICU [4–8]. Physicians often lack awareness of their patients’ wishes to avoid resuscitation in hospital [9]. Unwanted aggressive care at the end of life (EOL) is associated with increased healthcare costs, worse quality of life, and a worse death [10].

Decision-making about goals of care (GoC) has been defined as a communication process that occurs between clinicians and a patient to establish a care plan, and includes decisions about the use or non-use of life-sustaining treatments [11]. Less than a third of patients with advanced cancer have had a GoC discussion with a member of care team [10, 12]. As a result, patients may be subject to unwanted aggressive treatments near the end of life. Cancer patients who die in hospital experience more pain, anxiety, and physical and emotional distress compared to those who die at home with hospice services [13, 14]. Furthermore, when patients die in the ICU or in hospital, family members and caregivers are at higher risk for both post-traumatic stress disorder and prolonged grief disorder [15, 16]. In contrast, patients who have a more accurate understanding of their prognosis are more likely to decline aggressive interventions and resuscitation at the EOL [15]. GoC discussions between patients and physicians are associated with fewer life-sustaining interventions, fewer ICU admissions, better patient outcomes [14], and lower healthcare costs [17].

Hospitalization of a patient with advanced cancer marks an important inflection point in their illness trajectory and presents an important opportunity to clarify GoC. While the importance of support and communication around end-of-life decision-making in advanced cancer has been recognized [18], relatively few studies have identified the relative importance of barriers to GoC discussions in the oncology population. Previous studies have attempted to explore barriers experienced by physicians such as personal discomfort with death and dying [19, 20]. An improved understanding of the barriers to GoC discussions with hospitalized patients who have advanced cancer will inform the design of future strategies aimed at improving both the quantity and quality of GoC discussions in this patient population. The aim of this study was to identify barriers to GoC discussions with hospitalized patients who have advanced cancer, as perceived by oncology clinicians.

## Methods

We conducted a cross-sectional survey on all oncology wards of the Juravinski Hospital and Cancer Centre in Hamilton, Ontario, between July 2013 and May 2014. We defined oncology inpatient wards as a unit where staff oncologists, oncology residents, and nurses provided care for admitted patients with cancer. The Hamilton Integrated Research Ethics Board approved the study. Participants gave implicit informed consent through completion of the self-administered questionnaire in response to an invitation to participate voluntarily.

We surveyed oncology clinicians according to the following inclusion criteria: 1) staff oncologists providing care to cancer inpatients, 2) residents enrolled as a subspecialty trainee in the hematology, radiation, and medical oncology programs at McMaster University, and 3) nurses (registered nurses, advanced practice nurses, licensed practical nurses, or registered practical nurses) employed full time, or part time, on the oncology wards.

Physician-specific (staff oncologist or oncology resident) and nurse-specific versions of the questionnaire were created to capture items specific to each professional group. The questionnaire used in this study was adapted from a questionnaire used previously to quantify barriers to goals of care discussions, as perceived by clinicians, with seriously ill hospitalized patients on medical teaching units [21]. This original questionnaire was drafted by the authors, presented to a national focus group, revised by front-line clinicians, and then pilot-tested with physicians and nurses on a medical teaching unit (MTU) in Hamilton, Ontario, Canada. To adapt the survey for this study, oncologists provided clinical expertise and feedback on the content and structure of the questionnaire, including the hypothetical patient vignettes. We pilot-tested the initial adapted version with the study investigators and made revisions to improve clarity in a final version.

**Fig. 1:**
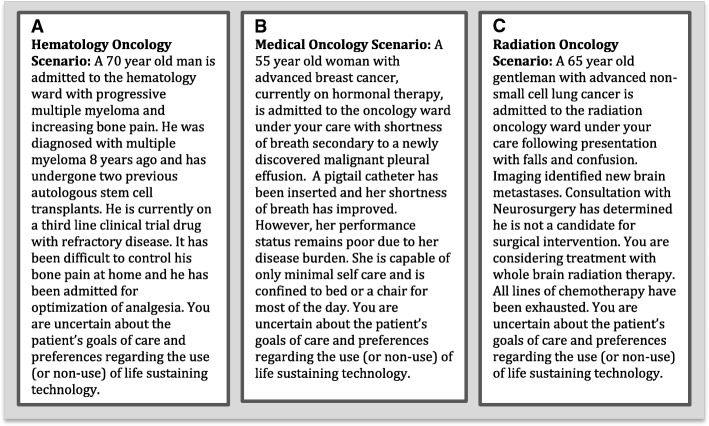
Clinical Vignettes. Clinical vignettes for clinicians in: (**a)** hematology oncology, (**b**) medical oncology, and (**c**) radiation oncology

The final questionnaire was then distributed in a paper-based format to oncology clinicians and electronic reminders were given to non-responders [22]. The preamble to the questionnaire presented the definition of goals-of-care discussions to be used for this study: “We define communication and decision-making about goals of care as a conversation in which, ideally, a patient or family member and the healthcare team establish the goals of treatment (e.g., cure, prolongation of life, comfort) and agree upon the types of life sustaining technology that will (or will not) be used to achieve those goals (e.g., CPR, mechanical ventilation, dialysis, intensive care unit admission, feeding tubes, or intravenous hydration)” [11]. Each questionnaire contained a clinical vignette (Fig. 1) that was tailored to each subspecialty of oncology (medical, hematology, radiation). Barriers to GoC discussions were assessed on a 7-point Likert scale (1 = extremely unimportant, 2 = very unimportant, 3 = somewhat unimportant, 4 = neither important nor unimportant, 5 = somewhat important, 6 = very important, 7 = extremely important). Participants were then asked to rate their willingness to participate in different aspects of GoC discussions (initiate discussion, exchange information, act as a decision coach, make a final decision) on the same 7-point Likert scale. Finally, participants were asked to rate how acceptable they found it to have other healthcare professionals initiate and participate in these different aspects of GoC discussions using the same 7-point Likert Scale.

All statistical analyses were performed with the use of SPSS software. We summarized continuous data using means and categorical data using proportions. We report survey responses as means and 95% confidence intervals.

## Results

**Fig. 2:**
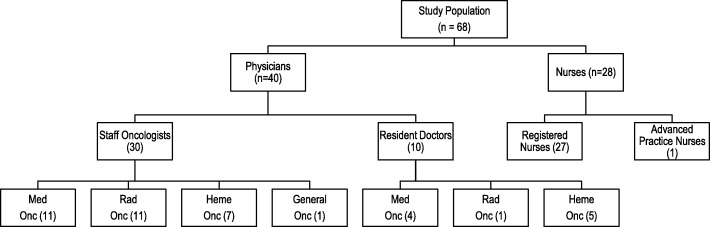
Study Population

The overall response rate was 68/185 (37%); (30/51 [57%] among staff oncologists, 10/26 [38%] among oncology residents, and 28/108 [26%] among oncology nurses). Figure 2 depicts the professional training of the study respondents, and the demographic characteristics of the study participants are shown in Table 1.

**Table 1:** Characteristics of Respondents

Nurses (*n* = 28)	
Age (mean)	40.2
Male	2 (7%)
Female	26 (93%)
Years of practice (mean)	12.0
Staff Physicians (*n* = 30)	
Age (mean)	42
Male	21 (70%)
Female	9 (30%)
Years of practice (mean)	12.5
Resident Physicians (*n* = 10)	
Age (mean)	31.4
Male	8 (80%)
Female	2 (20%)
Weeks of inpatient subspecialty oncology completed (mean)	5.9

### Barriers to goals of care discussions as perceived by oncology clinicians

**Fig. 3:**
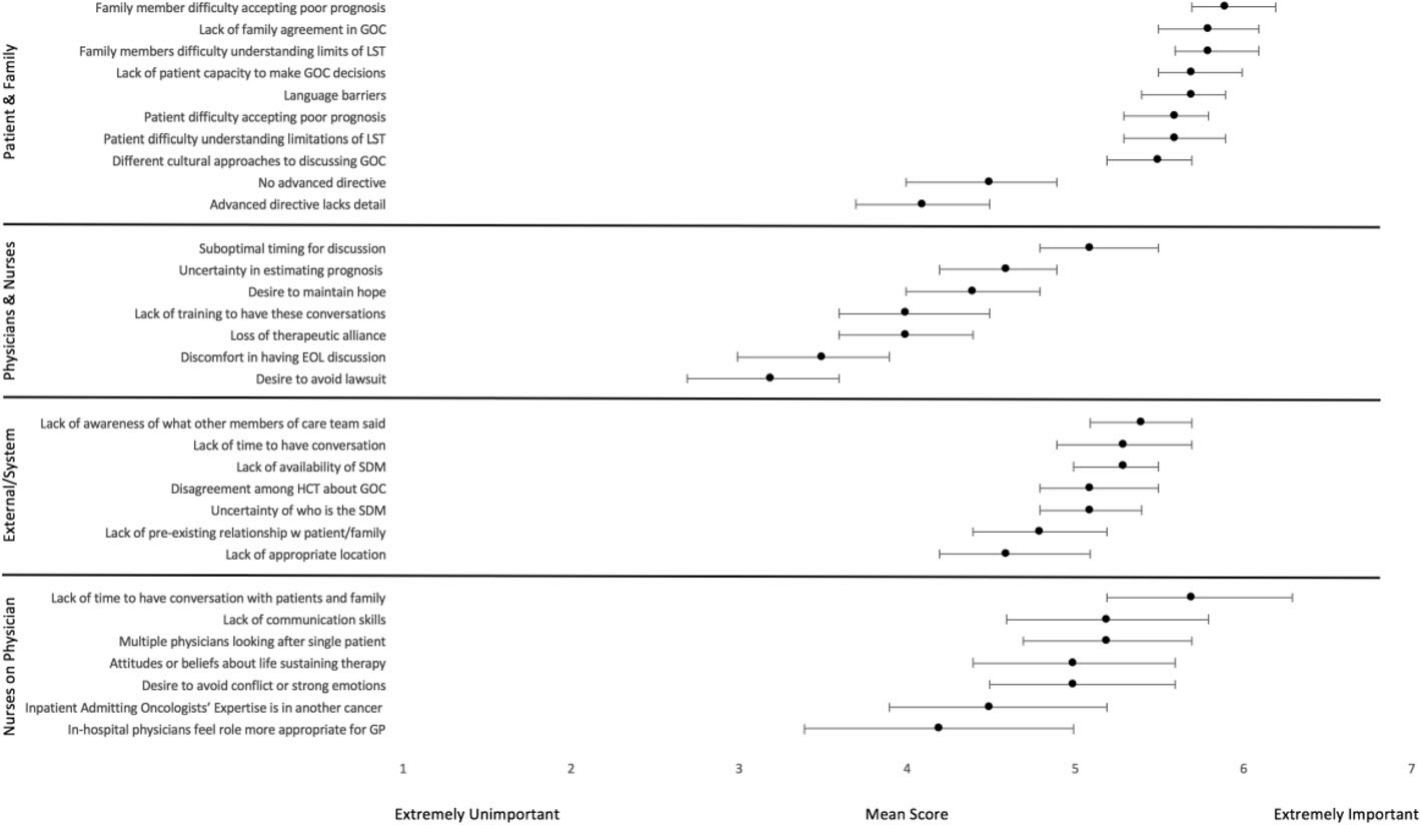
Perceived Barriers to Goals of Care Discussions. Combined mean scores from physicians and nurses on a Likert scale, rating the perceived barriers related to patient and family, the role of physicians and nurses from their own perspective, system and external factors, and the role of physicians from nurses’ perspective. The error bars denote 95% confidence intervals

Participants perceived patient and family member factors as the most important barriers to GoC discussions. These included: family members’ difficulty accepting a poor prognosis, lack of family agreement in the goals of care, difficulty understanding the limitations of life-sustaining treatments, lack of patients’ capacity to make goals of care decisions, and language barriers. Study participants viewed system factors and healthcare provider factors as less important barriers. Among external and system factors, lack of time was perceived as one of the more important barriers. Figure 3 illustrates the perceived barriers rated in this study, and all mean scores are tabulated in Additional file 1.

### Perceptions of inter-professional roles in communication and decision making about goals of care

Physicians and nurses together expressed a willingness to engage in GoC discussions, including initiating discussions with patients and family, exchanging important information such as diagnosis and prognosis, acting as a decision coach, and making a final decision together with the patient (Additional file 2).

**Fig. 4:**
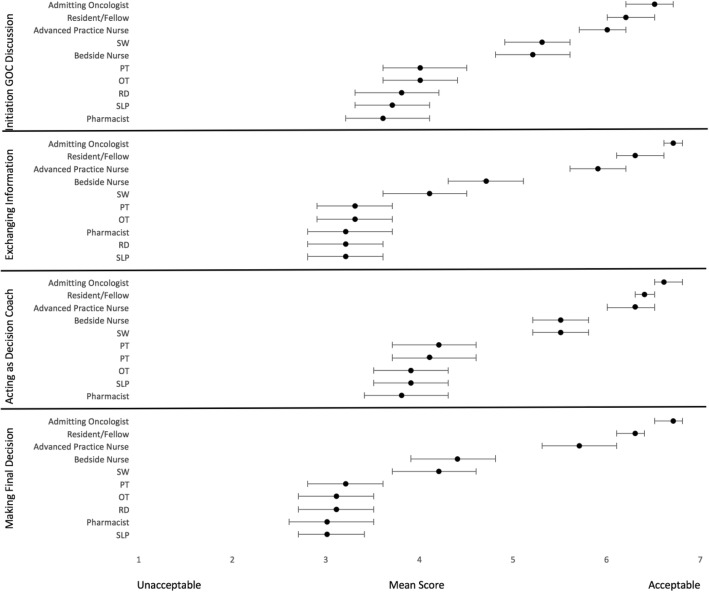
Acceptability of Inter-Professional Healthcare Team Members to Engage in Goals of Care Discussions. Combined mean score from physicians and nurses on a Likert scale rating the acceptability of inter-professional healthcare team members in initiating GOC discussion, exchanging information, acting as decision coach, making final decision. The error bars denote 95% confidence intervals

Participants were agreeable to involving allied health professionals in certain aspects of GoC discussions. For initiating a discussion or acting as a decision coach, participants felt that it would be appropriate for many different members of the inter-professional team to be involved, including the admitting oncologist, resident, advanced practice nurse, social worker, or bedside nurse. In contrast, for exchanging pertinent information such as diagnosis or prognosis, or making a final decision together with the patient, study participants felt that the most acceptable individual was the admitting oncologist, followed by the resident, and then advanced practice nurse. Participants felt it was less acceptable for other allied health professionals such as physiotherapists, occupational therapists, registered dieticians, speech language pathologists or pharmacists to engage in these other aspects of GoC discussions (Fig. 4, Additional file 3).

## Discussion

In this survey of staff oncologists, oncology residents, and nurses caring for patients with cancer at an academic cancer centre, we found that oncology clinicians perceived patient and family factors as the most important barriers to GoC discussions, and that system and healthcare provider factors were viewed as less important. Study participants also expressed a willingness to engage in many aspects of GoC discussions, such as initiating discussions, exchanging critical information such as prognosis, and making a final decision. Study participants found it acceptable for many different professional groups, including social workers and nurses, to initiate GoC discussions and act as a decision coach.

Cancer patients are more likely to receive care consistent with their preferences when they have had a GoC discussion with their physician, and are more likely to opt for symptom-directed care when they recognize their illness is terminal [23]. While GoC discussions improve the dying experience for patients [24], this study adds to the literature demonstrating that patient and family factors are the biggest barriers to effective GoC discussions as perceived by clinicians [21]. Although study participants rated practitioner factors as less important barriers to GoC, this has several interpretations and implications. First, it reflects that patients who have advanced cancer and their families may often find it difficult to confront a poor prognosis. As a result, oncology clinicians should anticipate that patients and families may find GoC conversations difficult or emotionally laden and be prepared to guide patients and families through these discussions. However patients who have cancer report that physicians are particularly inadequate in discussing emotional symptoms, life support preferences and hospice care, regardless of the patient’s age or disease stage [25]. Indeed, other studies have found that oncology practitioners often lack communication skills training and have discomfort with difficult discussions that include mortality acknowledgement and the introduction of a palliative approach to care [26–29]. This suggests that oncology clinicians may benefit from further training and continuing professional development to enhance their communication skills in order to best support patients and their families in GoC conversations [30]. Patients facing serious illnesses prefer to actively participate in EOL care planning [31–34]. While patient factors are often rated as the most significant barriers to communication, with formal training, oncology clinicians can master communication skills that will empower them to guide patients with advanced cancer through frank discussions about their prognosis and preferences for care at the end of life, preferably early in the course of disease [35, 36]. Formal training would also give clinicians an opportunity to learn and use validated tools for initiating these discussions [37] and develop an approach that recognizes the benefits of these conversations, while helping patients to discuss their values and preferences [19].

Despite many highly-rated barriers to GoC discussions, oncology physicians, residents and nurses described themselves as willing to engage in these important conversations. This likely reflects progressive awareness among providers that many cancer patients have not had GoC discussions, even at the time of admission to hospital, and of those who have, as many as half lack formal documentation of these conversations [9]. There is growing appreciation for both the importance and the timing of GoC discussions, as cancer patients who discuss their GoC with their healthcare team enter hospice care earlier, have fewer critical care admissions, a greater likelihood of dying outside the hospital, and a significantly improved quality of life [14, 38]. Finally, it likely reflects a rising awareness of advanced cancer patients wishing to avoid unwanted life support as a key element of quality end of life care [39].

The findings of this study also emphasized a lack of time as an important barrier, across all categories. Prioritizing protected time for important discussions is of great importance [40]. An acute admission to hospital marks an important change in disease trajectory, but only half of patients with advanced cancer who have documented EOL care discussions with their providers do so in the context of an emergency room visit or inpatient hospitalization [41]. Furthermore, acute care hospitals have performed poorly overall when evaluated on the quality of their end-of-life communication, as measured by validated quality indicators [42]. Ultimately, hospitalizations leave little time for discussion unless it is a priority, and patients may not have the same ability to recognize their hospitalization as a worsening of their prognosis. An admission to acute care often represents an important inflection point in a patient’s illness trajectory and should serve as a potent reminder to oncology clinicians to open the lines of communication and to discuss or revisit goals of care with their patients.

To further support patients and families, many tools and decision aids for patients have been explored, with the intention of helping patients ask questions, understand difficult decisions, and ultimately receive care that is aligned with their values and goals [43]. Question prompt lists have assisted patients and their caregivers to ask questions about end-of-life issues without contributing anxiety or impairing satisfaction [44]. Video decision aids have significantly decreased the likelihood that patients will opt for CPR and resuscitation, and be more certain of their decision-making [45, 46]. More significantly, decision aids have increased the likelihood that their choices ultimately reflect their own values [47]. While these tools are promising, the results of this study bring to light a myriad of barriers perceived by healthcare providers that need to be addressed. While decision aids may be helpful adjuncts, they cannot personalize disclosure of prognosis, and do not take the place of an honest, nuanced, and compassionate interaction between the patient and the clinicians caring for them.

Another implication of this study is the opportunity to include allied health professionals in GoC discussions. Second to physicians, study participants felt advanced practice nurses were most appropriate to engage in GoC discussions. Similarly, bedside nurses and social workers were felt to be acceptable individuals to specifically initiate discussions and act as a decision coach. This is supported by literature, where both nurses and social workers are significantly involved in end of life care, including a willingness to participate in GoC decision-making [48]. Social workers can be integral in information giving and education, as well as helping patients reach final decisions [49]. There is also great initiative and willingness on the part of other allied health members to play an active role [50–52]. These skilled individuals have a unique understanding of a patients’ health status, and concurrently can establish a unique rapport. This study’s findings suggest that there is an opportunity to adopt a more interprofessional approach to having conversations about a patient’s values, preferences, and goals of care.

There are several limitations to this study. It was conducted at a single academic cancer centre, and the respondents, on average, were relatively young or, in the case of oncology residents, had relatively little experience in subspecialty oncology settings. In addition, to the extent that non-responders held different views than our study participants, the 37% response rate in our study may limit the generalizability of our findings. However, our findings were remarkably similar to findings from a multi-center survey of practitioners (response rate 78%) that we conducted on hospital medical wards [21]. Therefore, our study confirms that the findings of our earlier study are generalizable to the oncology setting, suggesting that barriers to goals-of-care discussions during serious illness may be similar regardless of the specific disease state and that disease-agnostic interventions to improve communication during serious illness may be useful. Finally, it is possible that respondents might have been influenced by infrequent but very memorable interactions with advanced cancer patients or their families. Certain challenging interactions are subject to recall bias and as a result, respondents may have placed undue weight on patient and family factors.

## Conclusions

Oncology clinicians perceive patient and family factors as the most important barriers to GoC discussions. This insight forms an important platform for future interventions. Our findings highlight the potential for high levels of anxiety or even denial faced by patients with advanced cancer. This underscores the need for oncology clinicians to be equipped with strong communication skills to help patients and their families to navigate GoC decisions. Making time for these crucial conversations and planning to have them throughout the course of illness is imperative. GoC discussions should be initiated by physicians and nurses who have received focused training in this regard, and the discussion would be made richer with the help of skilled allied health professionals.

## Additional files

Additional file 1: Mean scores from physicians and nurses on a Likert scale, rating barriers related to patient and family factors, the role of physicians and nurses from their own perspective, system and external factors, and the role of physicians from nurses’ perspective. (DOCX 19 kb)

Additional file 2: Mean Likert scores, physicians and nurses rating their own willingness to engage in goals of care discussions. (DOCX 50 kb)

Additional file 3: Mean scores from physicians and nurses on a Likert scale, rating their perceptions of inter-professional roles in communicating and decision-making around goals of care. (DOCX 91 kb)

## Abbreviations

EOL: End of Life
ER: Emergency Room
GoC: Goals of Care
ICU: Intensive Care Unit
MTU: Medical Teaching Unit
