# Supplementary material for: Breaking silence: a survey of barriers to goals of care discussions from the perspective of oncology practitioners
Source: BMC Cancer. 2019 Feb 8;19:130. doi: 10.1186/s12885-019-5333-x (PMC6368724; doi:10.1186/s12885-019-5333-x)
Supplement: Supplementary file 1 — Mean scores from physicians and nurses on a Likert scale, rating barriers related to patient and family factors, the role of physicians and nurses from their own perspective, system and external factors, and the role of physicians from nurses’ perspective. (DOCX 19 kb) [file 12885_2019_5333_MOESM1_ESM.docx]

**Additional File 1**

A. Mean scores from physicians and nurses on a Likert scale, rating barriers related to patient and family factors, the role of physicians and nurses from their own perspective, system and external factors, and the role of physicians from nurses’ perspective.

| **Barriers Related to Patient and Family** | | |
| --- | --- | --- |
|  | **Mean** | **95% CI** |
| Family members difficulty accepting poor prognosis | 5.9 | 5.7, 6.2 |
| Lack of family agreement in GOC | 5.8 | 5.5, 6.1 |
| Family members difficulty understanding limits of LST | 5.8 | 5.6, 6.1 |
| Lack of patient capacity to make GOC decisions | 5.7 | 5.5, 6.0 |
| Language barriers | 5.7 | 5.4, 5.9 |
| Patient difficulty accepting poor prognosis | 5.6 | 5.3, 5.8 |
| Patient difficulty understanding limitations of LST | 5.6 | 5.3,5.9 |
| Different cultural approaches to discussing GOC | 5.5 | 5.2, 5.7 |
| No advanced Directive | 4.5 | 4.0, 4.9 |
| Advanced directive lacks detail | 4.1 | 3.7, 4.5 |
| **Barrier Related to the Role of Physicians and Nurses in Their Own Perspective** | | |
|  | **Mean** | **95% CI** |
| Suboptimal Timing for Discussion | 5.1 | 4.8, 5.5 |
| Uncertainty in Estimating Prognosis | 4.6 | 4.2, 4.9 |
| Desire to maintain hope | 4.4 | 4.0, 4.8 |
| Lack of Training to Have These Conversations | 4.0 | 3.6, 4.5 |
| Loss of therapeutic alliance | 4.0 | 3.6, 4.4 |
| Discomfort in having EOL discussion | 3.5 | 3.0, 3.9 |
| Desire to avoid lawsuit | 3.2 | 2.7, 3.6 |
| **Barriers related to the System or External factors** | | |
|  | **Mean** | **95% CI** |
| Lack of awareness of what other members of care team said | 5.4 | 5.1, 5.7 |
| Lack of time to have conversation | 5.3 | 4.9, 5.7 |
| Lack of availability of SDM | 5.3 | 5.0, 5.5 |
| Disagreement among HCT about GOC | 5.1 | 4.8, 5.5 |
| Uncertainty of who is the SDM | 5.1 | 4.8, 5.4 |
| Lack of pre-existing relationship w patient/family | 4.8 | 4.4, 5.2 |
| Lack of appropriate location | 4.6 | 4.2, 5.1 |
| **Barriers Related to Physician roles (Nurses Only)** | | |
|  | **Mean** | **95% CI** |
| Lack of time | 5.7 | 5.2, 6.3 |
| Lack of communication skills | 5.2 | 4.6, 5.8 |
| Multiple physicians looking after single patient | 5.2 | 4.7, 5.7 |
| Attitudes or beliefs about life sustaining therapy | 5.0 | 4.4, 5.6 |
| Desire to avoid conflict or strong emotions | 5.0 | 4.5, 5.6 |
| Inpatient Admitting Oncologists’ Expertise is in another cancer | 4.5 | 3.9, 5.2 |
| In-hospital physicians feel role more appropriate for GP | 4.2 | 3.4, 5.0 |
