# Supplementary material for: Breaking silence: a survey of barriers to goals of care discussions from the perspective of oncology practitioners
Source: BMC Cancer. 2019 Feb 8;19:130. doi: 10.1186/s12885-019-5333-x (PMC6368724; doi:10.1186/s12885-019-5333-x)
Supplement: Supplementary file 2 — Mean Likert scores, physicians and nurses rating their own willingness to engage in goals of care discussions. (DOCX 50 kb) [file 12885_2019_5333_MOESM2_ESM.docx]

**Additional File 2**

Mean Likert scores, physicians and nurses rating their own willingness to engage in goals of care discussions.

| **Action** | **Nurses** | | **Physicians** | | **Nurses and Physicians** | |
| --- | --- | --- | --- | --- | --- | --- |
|  | **Willingness** | **95%CI** | **Willingness** | **95%CI** | **Willingness** | **95%CI** |
| Initiate Discussions with Patients and Family | 4.9 | 4.3, 5.5 | 6.3 | 6.1, 6.5 | 5.8 | 5.4, 6.1 |
| Exchange Information (prognosis, diagnosis) | 4.7 | 4.0, 5.3 | 6.3 | 6.0, 6.6 | 5.6 | 5.3, 6.0 |
| Act As Decision Coach | 5.1 | 4.6, 5.7 | 5.9 | 5.5, 6.2 | 5.5 | 5.3, 5.8 |
| Make Final Decision | 4.5 | 3.9, 5.2 | 6.3 | 6.1, 6.5 | 5.6 | 5.2, 5.9 |
