# Supplementary material for: Breaking silence: a survey of barriers to goals of care discussions from the perspective of oncology practitioners
Source: BMC Cancer. 2019 Feb 8;19:130. doi: 10.1186/s12885-019-5333-x (PMC6368724; doi:10.1186/s12885-019-5333-x)
Supplement: Supplementary file 3 — Mean scores from physicians and nurses on a Likert scale, rating their perceptions of inter-professional roles in communicating and decision-making around goals of care. (DOCX 91 kb) [file 12885_2019_5333_MOESM3_ESM.docx]

**Additional File 3**

Mean scores from physicians and nurses on a Likert scale, rating their perceptions of inter-professional roles in communicating and decision-making around goals of care.

| **How acceptable is it for the following individuals to initiated GOC discussion?** | | | |
| --- | --- | --- | --- |
|  | **Mean** | **95% CI** |  |
| Admitting Oncologist | 6.5 | 6.2, 6.7 |  |
| Resident/Fellow | 6.2 | 6.0, 6.5 |  |
| Advanced Practice Nurse | 6.0 | 5.7, 6.2 |  |
| SW | 5.3 | 4.9, 5.6 |  |
| Bedside Nurse | 5.2 | 4.8, 5.6 |  |
| PT | 4.0 | 3.6, 4.5 |  |
| OT | 4.0 | 3.6, 4.4 |  |
| RD | 3.8 | 3.3, 4.2 |  |
| SLP | 3.7 | 3.3, 4.1 |  |
| Pharmacist | 3.6 | 3.2, 4.1 |  |
| **How acceptable is it for the following individuals to exchange information (diagnosis, prognosis)?** | | | |
|  | **Mean** | **95% CI** |  |
| Admitting Oncologist | 6.7 | 6.6, 6.8 |  |
| Resident/Fellow | 6.3 | 6.1, 6.6 |  |
| Advanced practice nurse | 5.9 | 5.6, 6.2 |  |
| Bedside nurse | 4.7 | 4.3, 5.1 |  |
| SW | 4.1 | 3.6, 4.5 |  |
| PT | 3.3 | 2.9, 3.7 |  |
| OT | 3.3 | 2.9, 3.7 |  |
| Pharmacist | 3.2 | 2.8, 3.7 |  |
| RD | 3.2 | 2.8, 3.6 |  |
| SLP | 3.2 | 2.8, 3.6 |  |
| **How acceptable is it for the following individuals to act as a decision coach?** | | | |
|  | **Mean** | **95% CI** |  |
| Admitting Oncologist | 6.6 | 6.5, 6.8 |  |
| Resident/Fellow | 6.4 | 6.3, 6.5 |  |
| Advanced practice nurse | 6.3 | 6.0, 6.5 |  |
| Bedside nurse | 5.5 | 5.2, 5.8 |  |
| SW | 5.5 | 5.2, 5.8 |  |
| PT | 4.2 | 3.7, 4.6 |  |
| OT | 4.1 | 3.7, 4.6 |  |
| RD | 3.9 | 3.5, 4.3 |  |
| SLP | 3.9 | 3.5, 4.3 |  |
| Pharmacist | 3.8 | 3.4, 4.3 |  |
| **How acceptable is it for the following individuals to make a final decision about goals of care?** | | | |
|  | **Mean** | **95% CI** |  |
| Admitting oncologist | 6.7 | 6.5, 6.8 |  |
| Resident/Fellow | 6.3 | 6.1, 6.4 |  |
| Advanced Practice Nurse | 5.7 | 5.3, 6.1 |  |
| Bedside Nurse | 4.4 | 3.9, 4.8 |  |
| SW | 4.2 | 3.7, 4.6 |  |
| PT | 3.2 | 2.8, 3.6 |  |
| OT | 3.1 | 2.7, 3.5 |  |
| RD | 3.1 | 2.7, 3.5 |  |
| Pharmacist | 3.0 | 2.6, 3.5 |  |
| SLP | 3.0 | 2.7, 3.4 |  |
